# Biological Characteristics and Whole-Genome Analysis of a Porcine *E. coli* Phage

**DOI:** 10.3390/vetsci12010057

**Published:** 2025-01-14

**Authors:** Shenghui Wan, Nana Li, Sajid Habib, Pei Zheng, Yanfang Li, Yan Liang, Yonggang Qu

**Affiliations:** 1 College of Animal Science and Technology, Shihezi University, Shihezi 832003, China; wanshenghui0807@foxmail.com (S.W.); linana1217@foxmail.com (N.L.); sajidhabib18@gmail.com (S.H.); yanfangli@shzu.edu.cn (Y.L.); liangyan@shzu.edu.cn (Y.L.); 2 Xinjiang Tecon Animal Husbandry Technology Co., Ltd., Changji 831399, China

**Keywords:** *Escherichia coli*, phage, biological characteristics, whole-genome analysis

## Abstract

(1) Background: In recent years, the increasing emergence of multidrug-resistant pathogens in pig farms has begun to pose a severe threat to animal welfare and, by extension, public health. In this study, we aimed to explore the biological characteristics and genomic features of bacteriophages that are capable of lysing porcine multidrug-resistant *E. coli*, which was isolated from sewage. In doing so, we provided a reference for phage therapies that can be used to treat multidrug-resistant strains. (2) Method: Using the multidrug-resistant *E. coli* isolate sq-1 as the host bacterium, bacteriophages were isolated and purified from fecal samples using a double-layer agar plate method. The morphology was observed using a transmission electron microscope, and its host range, optimal multiplicity of infection (MOI), one-step growth curve, thermal stability, acid–base tolerance, and in vitro antibacterial ability were tested. Genomic features were analyzed using whole-genome sequencing. (3) Results: A lytic phage named vB_EcoS_Psq-1 (abbreviated as Psq-1) was successfully isolated. Electron microscopy revealed that Psq-1 belongs to the family of long-tailed phages, possessing clear and transparent plaques of approximately 1 mm in diameter. Psq-1 only lyses the host bacterium and does not affect other *E. coli* strains or other species of bacteria. The optimal MOI for phage Psq-1 was 0.1, with a latent period of 25 min, an exponential growth period of 25 min, and a lysis yield of 44.21 PFU/cell. Its activity remains stable at temperatures between 40 °C and 60 °C and from pH 4.0 to pH 13.0. Psq-1 exhibited a significant inhibitory effect on *E. coli* in liquid culture medium. The nucleic acid type of phage Psq-1 was dsDNA, with a total genome length of 44,183 bp and a GC content of 52.16%. No known resistance, lysogenic, or virulence-related genes were detected. The whole genome contains 55 open reading frames (ORFs). (4) Conclusions: This study isolated a bacteriophage that is capable of lysing multidrug-resistant *E. coli*. Characterized by a narrow *E. coli* lysis range, a long latent period, limited lytic ability, and stable biological properties, this bacteriophage can serve as a reference isolate for *E. coli* phages and can provide biological materials and data to support research on bacteriophages that are effective against multidrug-resistant porcine *E. coli*.

## Simple Summary

In this study, we aimed to investigate the characteristics of bacteriophages that are capable of lysing multidrug-resistant *Escherichia coli* derived from pigs, providing a reference for phage therapy. Phages obtained from fecal waste were isolated and purified, and their morphology was observed using transmission electron microscopy. Their biological characteristics were identified and whole-genome sequencing analysis was performed. A bacteriophage of *Siphaviridae*, which lyses porcine *E. coli*, was isolated and named vB_EcoS_Psq-1 (Psq-1). It has a narrow lytic range but possesses stable biological properties. The genome of Psq-1 is dsDNA, which possesses a length of 44,183 bp and a GC content of 52.16%. It does not contain known resistance, lysogenic, or virulence genes and has 55 open reading frames (ORFs). Psq-1 exerted a significant inhibitory effect on *E. coli* during in vitro antibacterial experiments. Psq-1 can serve as a reference isolate, aiding in the study of bacteriophages that can be used to treat multidrug-resistant porcine *E. coli*.

## 1. Introduction

*Escherichia coli*, which belongs to the family *Enterobacteriaceae* and the genus *Escherichia* and is widely distributed in the intestines of both humans and animals, is the most common type of Gram-negative bacteria. As a result of environmental changes, some strains of *E. coli* gradually acquire certain virulence factors [1] and become pathogenic. These bacteria can be categorized as enteropathogenic or extraintestinal pathogenic *E. coli*. Such bacteria pose significant problems for the swine-farming industry, inducing edema, neonatal diarrhea, and white diarrhea in piglets, leading to high mortality rates. These illnesses not only affect the animals’ growth and development but also result in significant economic losses for pig-farming operations [2]. As the world’s largest producer and consumer of pork, our country has historically used significant quantities of antibiotics to prevent and control bacterial diseases within pig farms. Unfortunately, reports indicate that the widespread use of antibiotics over an extended period has accelerated the emergence and spread of antibiotic-resistant strains [3,4]. The widespread prevalence of multidrug-resistant bacterial strains has significantly reduced the effectiveness of antibiotics [5], and, thus, the search for safe and efficient treatment strategies to address antibiotic-resistant bacterial infections in animal husbandry is becoming increasingly urgent.

Phages—viruses that specifically infect and lyse bacteria—have widespread prevalence in water, soil, air, and feces [6]. They possess high host specificity, stable safety, and effectiveness in controlling antibiotic-resistant bacteria [7]. As such, they are viewed as a highly promising means of treating bacterial infections in the postantibiotic era. Phages have widespread applicability to various tasks, including disease treatment, food preservation, control of plant pathogens, vaccine development, delivery systems, management of bacterial biofilms, and surface disinfection [8]. D’Herelle [9] was the first researcher to test phage therapy in animals, successfully treating fowl typhoid in chickens—the survival rate of birds receiving the phage treatment was 95–100%, while the untreated control group had a survival rate of 0–25%. Meanwhile, in their work, Atterbury and colleagues [10] selected three lytic phages that had a broad host range against *Salmonella enteritidis*, *Citrobacter rodentium*, and *Salmonella typhimurium*. They demonstrated good preventive effects in 36-day-old Ross broilers. In our study, we isolated a lytic phage from multidrug-resistant *E. coli* derived from pigs and analyzed its biological and genomic characteristics. Through whole-genome sequencing, it is possible to compare the genes of phages with a broad lytic spectrum to those with a narrow lytic spectrum. This can help to identify the reasons for the differences in phages’ lytic ranges, providing a theoretical basis and data to support future phage therapies with an expanded host range.

## 2. Materials and Methods

### 2.1. Strain and Sample Collection

The multidrug-resistant *E. coli* strain sq-1 and other tested strains (30 strains of *E. coli*, 21 strains of *Klebsiella pneumoniae*, and two strains of *Proteus vulgaris*) were isolated and identified by the Animal Infectious Disease Laboratory at Shihezi University. In preliminary tests, the exponential growth phase of the *E. coli* strain sq-1 exhibited resistance to eight antibacterial agents: β-lactam antibiotics, including penicillin G, amoxicillin, and ampicillin; chloramphenicol-class antibiotics such as florfenicol; sulfonamides such as trimethoprim–sulfamethoxazole; tetracyclines such as oxytetracycline; fluoroquinolones, including enrofloxacin; and glycopeptides such as vancomycin. This bacterium is classified as a multidrug-resistant strain (unpublished data).

Feces and wastewater were collected from large-scale pig farms in the Ili region of Xinjiang and underwent subsequent phage isolation.

### 2.2. Main Reagents and Instruments

Luria–Bertani medium (Cat: HB0128) was purchased from Hopebio Co., Ltd. (Qingdao, China); agar powder (Cat: A8190), 2% Phosphotungstic acid negative staining solution (Cat: G1870), and PEG8000 (Cat: P8260) were obtained from Beijing Solarbio Science & Technology Co., Ltd. (Beijing, China). The antimicrobial disks were obtained from Hangzhou Binhao Microbial Reagent Co., Ltd. (Hangzhou, China), and the viral DNA/RNA extraction kits (Cat: S50622) were acquired from TransGen Biotech (Beijing, China).

The Pico17 centrifuge was purchased from Thermo-Fisher Scientific (Shanghai) Instruments Co., Ltd. (Shanghai, China), the BMJ-160 vertical constant-temperature incubator was obtained from American Genius (Suzhou) Co., Ltd. (Suzhou, China), and the HHW21.600ALL intelligent electric constant-temperature water bath was acquired from the Beijing Yongguangming Medical Instrument Factory (Beijing, China). The HT7700 transmission electron microscope was obtained from Hitachi High-Technologies Corporation (Tokyo, Japan). The IS-RDV1 vertical double-layer constant-temperature shaker was purchased from American Genius Co., Ltd. (Suzhou, China).

### 2.3. Enrichment of Phages

Phage enrichment was carried out as described by Nikkhahi et al. [11], with slight modifications. A culture of the log period *E. coli* strain sq-1 was used as the host bacterium, inoculated into LB liquid medium, and incubated overnight at 37 °C with shaking at 180 r/min. Samples of feces and wastewater collected from pig farms were mixed and soaked in SM buffer overnight for phage enrichment purposes. The following day, the mixture was filtered through gauze to remove any debris. A total of 40 mL of the filtered fecal suspension and 1 mL of the enriched *E. coli* culture were added to 300 mL of LB liquid medium, which was then incubated at 37 °C with shaking at 180 r/min for 12 h. The culture was centrifuged at 12,000 r/min for 15 min at 4 °C, and the supernatant was collected and filtered through a 0.22 μm filter for sterilization. The resulting filtrate was designated as the phage stock solution and was stored at 4 °C for future use.

### 2.4. Isolation and Purification of Phages

Chang HC et al. [12] employed a drop-by-drop technique to determine whether the phage stock solution contained lytic phages. A 100 μL log period *E. coli* culture was inoculated onto LB solid medium and evenly spread using an inoculation loop. After the culture was absorbed, 5 μL of the phage stock solution was added dropwise onto LB solid medium, which was then incubated at 37 °C for 8–12 h, after which the formation of lysis plaques was observed.

Using a sterile pipette tip, a single clear plaque was selected and placed in 200 μL of SM buffer solution, followed by incubation at 4 °C overnight. Afterward, the liquid was collected and added to 100 μL of host bacteria in LB broth, incubated, and shaken for 5–8 h. The culture was removed and centrifuged at 12,000 r/min for 15 min at 4 °C, and then 100 μL of the supernatant and 100 μL of the host bacteria were transferred into a test tube. Phages were purified using the double-layer agar plate method, and the purification process was repeated 3–5 times until uniform lytic plaques of similar size and shape appeared on the plates.

### 2.5. Concentration of Phages and Observation by Electron Microscopy

Referring to the methods of Hou Gongmingzhu et al. [13], a slight adjustment was made to the polyethylene glycol (PEG) precipitation technique used for concentrating purified phages. An appropriate amount of PEG8000 (to a final concentration of 10%) was added to the prepared phage solution and stirred until dissolved, and the solution was placed in an ice bath for 20–24 h to allow the phage particles to fully precipitate. All liquid was transferred to a 50 mL centrifuge tube and centrifuged at 4 °C at 8000 r/min for 10 min, and the supernatant was discarded. The phage pellet was resuspended in SM buffer and allowed to stand at 4 °C for 1 h. Subsequently, an equal volume of chloroform was added to extract PEG and cell debris from the phage solution. The mixture was centrifuged at 4 °C and 3000 r/min for 15 min to recover the hydrophilic phase containing the phage particles, yielding a concentrated phage solution.

The phage concentrate (50 μL) was added to the copper grid and allowed to adsorb for 10 min. Next, 2% phosphotungstic acid staining was implemented for 5 min, followed by removal of the copper grid. The sample was irradiated with infrared light for 30 min, and the phage morphology was observed using a transmission electron microscope.

### 2.6. Study of the Biological Characteristics of Phages

#### 2.6.1. Determination of Phage Lysis Profile

Using the drop method, the lytic range of the isolated bacteriophages against 30 strains of *E. coli*, 21 strains of *Klebsiella pneumoniae*, and two strains of *Proteus vulgaris*, excluding the host strain sq-1, was determined. Firstly, 200 µL of fresh bacterial solution was collected during the exponential growth phase (OD_600_ = 0.6) and evenly coated on LB solid medium. After drying, 5 μL of the bacteriophage (10^8^ PFU/mL) suspension was added to the inoculated area, and the plates were incubated at 37 °C overnight. The formation of plaques was observed; if plaques appeared while the control area showed normal bacterial growth, it indicated that the bacteriophage lysed the bacterial strain.

#### 2.6.2. Optimal Multiplicity of Infection and One-Step Growth Curve

To determine the optimal multiplicity of infection (MOI) of the bacteriophages, we utilized the method described by Li et al. [14], with slight modifications. The host bacteria were cultured to the logarithmic growth phase (OD_600_ = 0.6, about 10^8^ CFU/mL) and mixed with various MOIs (100, 10, 1, 0.1, 0.01, 0.001) in quantities of 100 μL each. Following the addition of 800 μL LB liquid medium, the mixture was incubated at 37 °C with shaking at 180 rpm for 4 h. After centrifugation at 12,000 r/min for 10 min, the supernatant was filtered through a 0.22 μm membrane to remove unlysed bacteria. The bacteriophage titer in the different treatment groups was assessed using the double-layer agar plate method, and the results were observed and counted after 12 h. The experiment was repeated thrice, and the average was calculated.

To determine the one-step growth curve of the bacteriophages, we utilized the method described by Hou Gongmingzhu et al. [13], with some adjustments. The bacteriophage solution was mixed in equal volumes with *E. coli* in the logarithmic growth phase at the optimal multiplicity of infection (MOI) ratio and incubated at 37 °C for 10 min for adsorption. The mixture was centrifuged at 12,000 r/min for 10 min, and the supernatant was discarded. After that, the pellet was resuspended in 20 mL LB liquid medium and incubated in a shaking incubator at 37 °C and 180 rpm. Five milliliters of the culture was collected at different time points (0, 5, 10, 15, 20, 25, 30, 40, 50, 60, 80, 100, 120, 150, and 180 min) and centrifuged at 12,000 r/min for 5 min, and the supernatant was used to determine the bacteriophage titer via the double-layer agar plate method. The experiments were repeated thrice, and the average value was calculated.

#### 2.6.3. Thermostability and pH Sensitivity

Two milliliters of phage solution was placed in a constant-temperature water bath at different temperatures (40 °C, 50 °C, 60 °C, 70 °C, and 80 °C). Samples of 200 μL were taken at incubation times of 20, 40, and 60 min. The phage titer was determined using the double-layer agar plate method. The experiment was repeated thrice, and the average values were calculated.

The phage solution (100 μL) was mixed with 900 μL of LB liquid culture media at different pH values (1.0, 2.0, 3.0, 4.0, 5.0, 6.0, 7.0, 8.0, 9.0, 10.0, 11.0, 12.0, and 13.0) and incubated in a 37 °C water bath for 2 h. After incubation, the phage titer was assessed using the double-layer agar plate method. The experiment was repeated three times, and the average value was calculated.

### 2.7. In Vitro Phage Inhibition Test

An in vitro phage inhibition test was conducted as described by Han et al. [15]. A single colony of the host bacterium sq-1 was inoculated on 5 mL LB medium and incubated to OD_600_ = 0.2, and phages were added at MOI = 10, 1, and 0.1. The mixture was incubated at 37 °C with shaking at 180 r/min in an oscillating incubator for 12 h. Phage-free *E. coli* sq-1 culture medium was used as the control. The absorbance (OD_600_) was measured every 1 h during the culture using a full-wavelength enzymograph. The experiment was repeated three times, and the average value was calculated.

### 2.8. Sequencing and Bioinformatics Analysis of Phage Genome

The phage genome was extracted according to the instructions of the viral DNA/RNA extraction kit, and the product was sent to Hangzhou Huitong Biotechnology Co., Ltd. (Hangzhou, China), for sequencing. The whole genome was sequenced using the Illumina NovaSeq 6000 sequencing platform, with the TruSeq RNA library preparation method and paired-end sequencing mode, and Newbler software (Version: 2.9.0) was utilized for sequence assembly. GeneMarkS software (Version: 4.6b, https://exon.gatech.edu/genemarks.cgi, accessed on 3 December 2023) was used to predict the gene sequences. The Antibiotic Resistance Gene Database (CARD) (https://card.mcmaster.ca, accessed on 3 December 2023) was used to predict the phage genome of antibiotic resistance genes, and online software (http://www.genomicepidemiology.org/services/, accessed on 7 October 2024) was utilized to ascertain the virulence factors of phage genome genes. The online software CGView Server (https://js.cgview.ca, accessed on 1 October 2024) was used to map the entire phage genome. The terminal large subunit protein, endolysin, and long-tail fibrin of the phages were used for phylogenetic tree analysis. The sequences of the terminal large subunit protein, endolysin, and long-tail fibrin of the phage were compared using the NCBI website, and the phylogenetic tree was drawn using MEGA 7.0. Finally, we used the online tool ESPript 3.0 (https://espript.ibcp.fr/ESPript/cgi-bin/ESPript.cgi, accessed on 31 December 2024) to compare the amino acid sequences of the phage long-tail fibrin proteins.

### 2.9. Statistical Analysis

All statistical analyses were performed using GraphPad Prism 9.0. The significance of the experimental data was assessed via multiple *t*-tests. The error bars represent the standard deviation (SD) of the mean.

## 3. Results

### 3.1. Isolation and Purification of Phage and Morphological Observation Under an Electron Microscope

**Figure 1:**
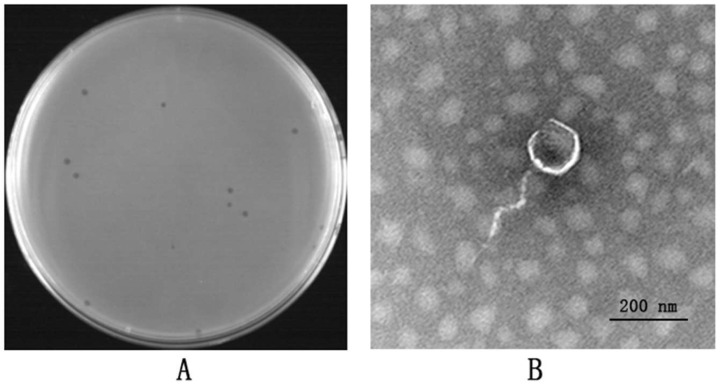
Morphological characteristics of phage Psq-1: (**A**) plaque morphology of phage Psq-1; (**B**) transmission electron microscope morphology of phage Psq-1 (50,000×).

The multidrug-resistant *E. coli* strain sq-1 was used as a host bacterium to successfully isolate a lytic *E. coli* phage from mixed samples of sewage and feces. According to the standards of the International Committee on Taxonomy of Viruses (ICTV) [16], the bacteriophage was named vB_EcoS_Psq-1 (Psq-1). It has neat and clear edges, with a diameter of approximately 1 mm, and no halo (Figure 1A). Transmission electron microscopy (TEM) revealed that Phage Psq-1 has a regular icosahedral head with a diameter of 76.60 nm and a tail of 127.66 nm in length. It conforms to the morphological characteristics of the order *Caudovirales* and the family *Siphoviridae* (Figure 1B).

### 3.2. Biological Characteristics of Phage Psq-1

#### 3.2.1. Host Range of Phage Psq-1

The bacteriophage Psq-1 can lyse the host bacterium sq-1; however, it is incapable of lysing the other *E. coli*, *Klebsiella pneumoniae*, and *Proteus mirabilis* from pigs.

#### 3.2.2. Optimal Multiplicity of Infection and One-Step Growth Curve of Phage Psq-1

**Figure 2:**
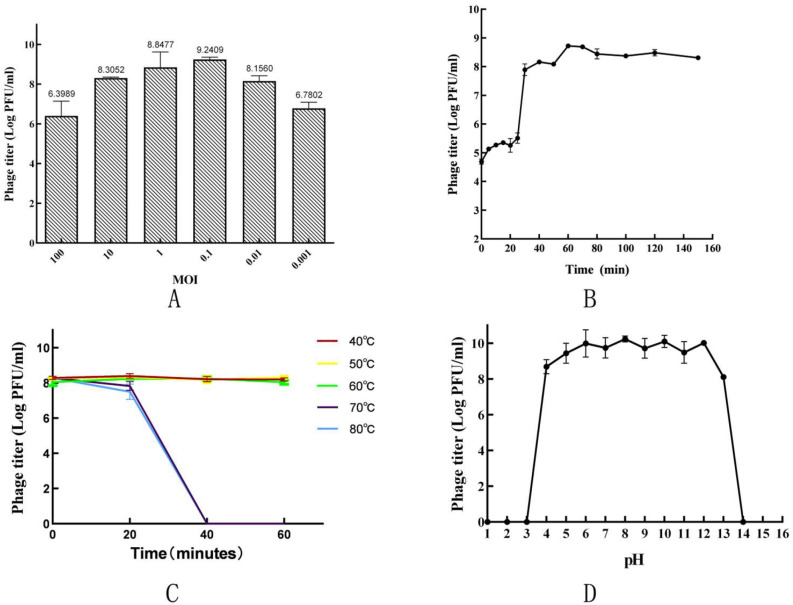
Biological characteristics of phage Psq-1: (**A**) determination of optimal multiplicity of infection (MOI) of phage Psq-1; (**B**) one-step growth curve of phage Psq-1 (50,000×); (**C**) thermal stability of phage Psq-1; (**D**) stability of phage Psq-1 at different pH values.

At a MOI of 0.1, phage Psq-1 had the highest titer of approximately 1.74 × 10^9^ PFU/mL (Figure 2A). The optimum MOI for this phage is 0.1.

The one-step growth curve showed that the latency period of Psq-1 was approximately 25 min, and the titer of Psq-1 increased rapidly within a 25–40 min period. Within 40–70 min, the phage titer slowly increased until the 70 min mark, at which point it stabilized, reaching 8.444 log PFU/mL (Figure 2B). The burst period of the phage was 15 min, and the cleavage volume was 44.21 PFU/cell.

#### 3.2.3. Thermostability and pH Sensitivity of Phage Psq-1

Phage Psq-1 was incubated at 40 °C, 50 °C, and 60 °C for 60 min, and the titer remained consistent. After incubation at 70 °C and 80 °C for 20 min, the phage titer decreased slightly; after 40 min of incubation, all phage activity ceased (Figure 2C).

At a pH of 4.0~13.0, the phage maintained high activity, whereas at pH 6.0 and 12.0, the phage titer decreased slightly. Finally, at pH ≤ 3.0 and pH ≥ 14.0, the phages were completely inactive (Figure 2D).

### 3.3. In Vitro Phage Psq-1 Inhibition Test

**Figure 3:**
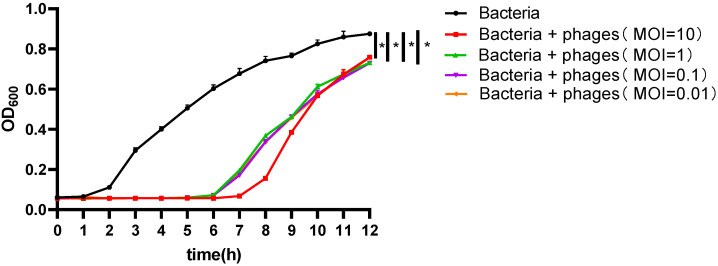
Phage Psq-1 in vitro inhibition assay (*: *p* < 0.05).

In the control group, to which no phage was added, *E. coli* Sq-1 continued to grow during the 12-h culture period. However, when phages at MOI = 10, 1, and 0.1 were added to the medium, phage Psq-1 significantly inhibited the growth of *E. coli* Sq-1 during the first 0 to 6 h. The OD values of the control group consistently increased, while the OD values of the test groups with phages exhibited no significant changes. Between 6 and 12 h, the OD values in the test groups continued to increase but remained below the OD values of the control group, indicating that phage Psq-1 can inhibit the growth of *E. coli* in vitro (Figure 3).

### 3.4. Whole-Genomic Analysis of Phage Psq-1

Whole-genome sequencing was performed, and the average sequencing depth was 17620X. The nucleic acid type of Psq-1 (GenBank accession: PQ595991) was double-stranded DNA (dsDNA), the total length of the genome was 44 183 bp, the GC content was 52.16%, and the AT content was 47.84%. CARD and the VirulenceFinder 2.0 database were compared, revealing that Phage Psq-1 did not carry known resistance or virulence genes.

**Figure 4:**
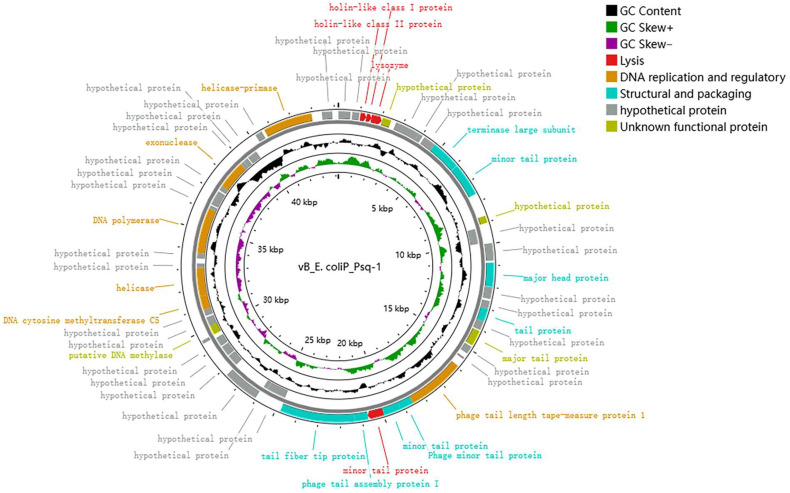
Whole-genome map of Phage Psq-1.

**Figure 5:**
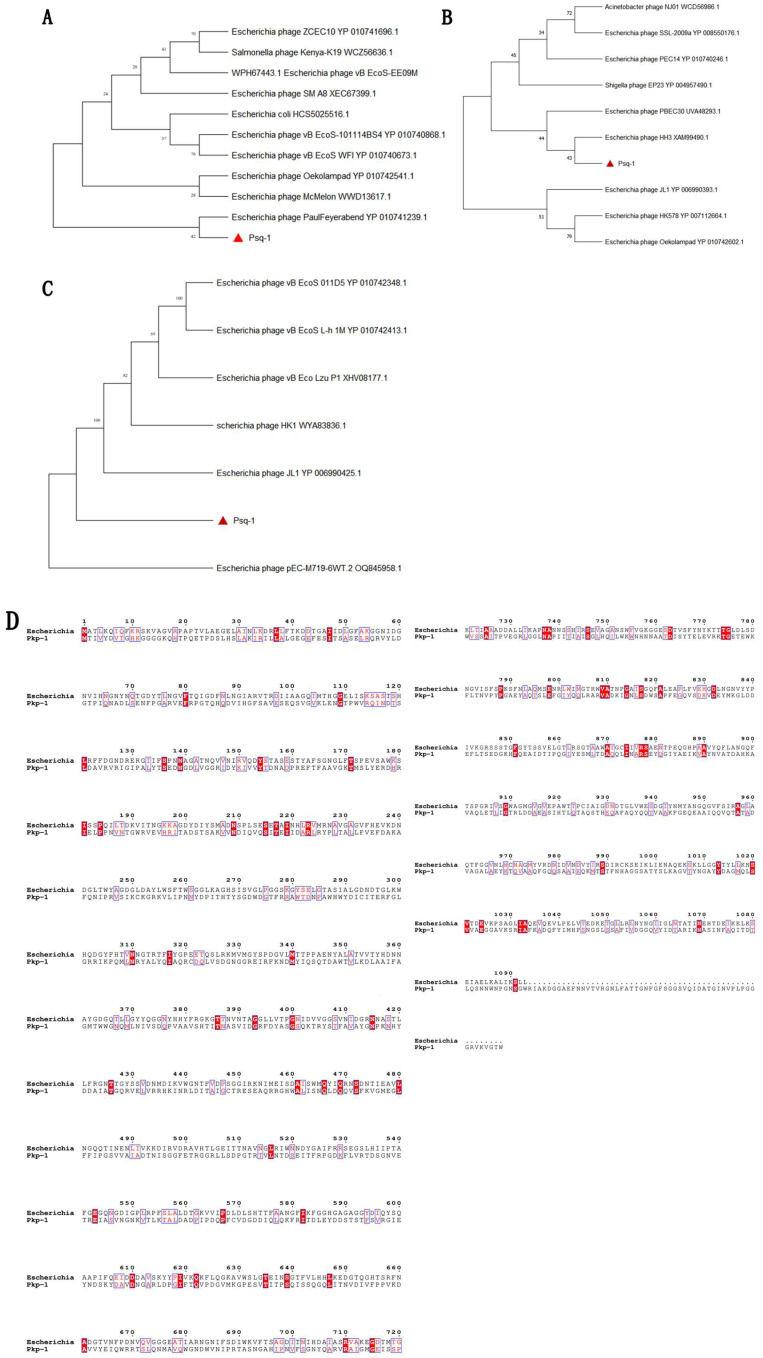
(**A**) Phylogenetic tree composed of terminal large subunits; (**B**) phylogenetic tree composed of endolysin; (**C**) phylogenetic tree composed of long-tail fibrin; (**D**) amino acid comparison of phage long tail fibrin.

According to bioinformatics analysis, there were 55 open reading frames (ORFs) in the whole genome of phage Psq-1, of which 34 were forward-coded and 21 were reverse-coded. Of these, 26 ORFs encoded known functional proteins, 29 were hypothesized protein sequences, and one was an unknown functional protein sequence (Table 1). The known functional proteins of phages can be divided into four functional blocks: structural and packaging genes, genes related to DNA replication and regulation, genes related to metabolism, and genes related to cleavage. The online tool CGview (https://js.cgview.ca; accessed on 3 December 2023) was used to visualize the phage whole-genome map. The outer open CDSs are painted in blue (phage structure and packaging proteins), red (lysis-related proteins), orange (DNA replication- and regulation-related proteins), and purple (metabolic-related proteins); triangular arrows indicate different functional module genes (Figure 4). Whole-genome analysis revealed that ORF3, ORF4, and ORF5 control the synthesis of proteins associated with bacteriophage lysis of bacteria. ORF3 and ORF4 are holin-like class II proteins, also known as perforin, which are similar to the holin proteins in bacteria. Perforins are a class of proteins that participate in bacteriophages’ infection of bacteria by forming holes in the bacterial cell membrane, helping the bacteriophage to release its genetic material into the bacterial cell interior. ORF5 encodes lysozyme, which can destroy mucopolysaccharides in the bacterial cell wall, causing the cell wall to rupture and, ultimately, leading to bacterial lysis. A phylogenetic tree was constructed with conserved terminal large subunit protein sequences of evolutionary significance (Figure 5), and phage Psq-1 and Escherichia phage *PaulFeyerabend* (GenBank accession: NC_073067.1) were the closest relatives. This suggests that phage Psq-1 belongs to the realm *Heunggongvirae*, the phylum *Uroviricota*, the *Caudoviricetes* class of tailed bacteriophages, and the family *Siphoviridae*, as a type of dsDNA virus. The phylogenetic tree was constructed using the neighbor-joining method with 1000 bootstrap replicates. The phylogenetic tree constructed using an endolysin protein sequence showed that phage Psq-1 was closely related to *Escherichia* phage HH3 (GenBank accession: XAM99490.1). The long-tail fibrin of Phage Psq-1 has a distant relationship with the other six types of long-tail fibrin. A comparison between the genome of phage Psq-1 and that of phage pEC-M719-6WT.2 (GenBank accession: OQ845958.1), which has a wide lytic range, revealed that the average similarity of its long-tail fibrin was 6.53%, with an overall similarity of 59.97%.

## 4. Discussion

Due to the widespread use of antibiotics, multidrug-resistant strains have emerged, posing a serious threat to the pig-farming industry and to human public health [2]. Aiming to reduce the use of antibiotics and explore biological agents for treating diseases caused by *E. coli* infections in pig farms, we collected sewage samples from large-scale pig farms and successfully isolated a phage Psq-1 that is capable of lysing *E. coli* strain sq-1. The biological characteristics, genomic features, and genetic background of Psq-1 were preliminarily explored, providing a theoretical basis for further research on phages. The phage Psq-1 isolated in this experiment belongs to the family *Siphoviridae* and possesses a long latency period and burst phase. A shorter latency period can enable phages to function more quickly and produce a large number of offspring. Phage Psq-1 has strong tolerance to a range of temperatures and pH levels and can survive in most environments. The optimal MOI of phage Psq-1 is consistent with the isolation of phage BP32 in a previous study by Wu et al. [17], but the latency period and burst phase are shorter than those of BP32. The lysis yield of phage Psq-1 is higher than that of vB-EcoC-P17 (17 PFU/cell) [18], but the lysis range of phage P17 is broader. In terms of temperature and pH tolerance, phage Psq-1 is stronger than phage SDYTW1-F1-2-2 and phage IME178 [19,20]. Different phages have different biological characteristics; however, safety is the priority in clinical practice. Phage Psq-1 was compared via CARD and VirulenceFinder2.0, and no known resistance or virulence genes were identified. The host range of this phage is relatively narrow, which may limit its clinical applicability. However, it still has various potential applications in specific environments. For example, in industrial or agricultural settings, where the temperature and pH are relatively stable, Psq-1 may be an effective biological control agent. In addition, its shorter latency period and burst phase indicate that it can respond quickly to bacterial infections and provide rapid therapeutic effects. To expand its applicability, Psq-1 can be combined with other phages to form a phage cocktail [21]. Phages, a type of antibacterial biological agent, are characterized by their specific and efficient lysis of host bacteria, safety, low cost, and abundant sources. Continuously enriching the phage library and comparing the differences between different phages facilitates the modification of alternative antibiotic biological agents with a wide lysis range and high lysis efficiency [22]. This not only expands the host range but also reduces the risk of bacterial resistance. Further genetic engineering modifications may enhance the characteristics and safety of Psq-1. Isolating and characterizing Psq-1 provides valuable information for the development of novel antibacterial strategies. In future, researchers should focus on evaluating its in vivo activity, safety, and synergistic effects with other treatment methods, which will inform its application in clinical practice.

Some research indicates [23] that, when exploring the antibacterial effects of bacteriophages in liquid environments, bacterial concentrations tend to rise in the later stages of inhibition, leading to a decrease in the bacteriophages’ effectiveness. In this study, we assessed the in vitro antibacterial effect of the *E. coli* phage Psq-1 and revealed that Psq-1 exhibits its strongest antibacterial effect against *E. coli* sq-1 within the first 6 h. This duration is notably shorter compared to the prolonged optimal antibacterial effect observed for the phages isolated by Liao Binru et al. [24]. During the experiment, we noted that, after a certain period, the bacterial concentration rebounded, indicating a decline in antibacterial efficacy. This finding is consistent with results reported by Rong Ruiyao [25], Man Cheng [26], and Chen Liying [27]. It can be inferred that *E. coli* sq-1 may be resistant to phage Psq-1, and, thus, the inhibitory effect of the phage decreased. When the MO = 10, the inhibitory effect of the bacteriophage is stronger compared to that observed at MOI values of 1, 0.1, and 0.01. Thus, using a higher MOI in clinical treatment may yield better therapeutic outcomes for this bacteriophage.

In this study, we predicted multiple important functional genes in the Psq-1 phage genome, including ORF3, ORF4, and ORF5, which are important lytic proteins of the phage, and holin, a membrane protein that can regulate the length of the infection cycle to ensure optimal lysis. Lysozyme acts as a lytic protein by degrading peptidoglycan in the bacterial cell walls and disrupting bacterial membranes [28]. At present, in addition to studying the biological characteristics of phages, researchers are expressing an increasing interest in the special protein lyases and depolymerases encoded by bacteriophages. Exploring the use of these two enzymes and their antibacterial effects when combined with other drugs is a current research hotspot [29]. ORF32 is a phage-long-tail fibrin that can bind to host surface receptor proteins, thereby initiating bacterial lysis [30]. Most regions of the long-tail fibrin of phages are relatively conserved, so different phages can change the size of their host range through the recombination of long-tail fibrin sequences [31]. This may also contribute to the relatively small host range of the Psq-1 phage. Whole-genome sequencing of this phage can be compared with other spectral phage sequences to determine the factors that control the phage lysis range, providing good biomaterial for future research on antibacterial biologics.

## 5. Conclusions

In this study, we isolated the bacteriophage Psq-1, of the *Siphoviridae* family, from sewage. Psq-1 only lyses host bacteria, leaving other strains of *E. coli* and other species of bacteria unaffected. The optimal multiplicity of infection (MOI) for phage Psq-1 is 0.1, and it has an incubation period of 25 min, a burst phase of 15 min, and a cleavage volume of 44.21 PFU/cell. Its activity remains stable in environments ranging from 40 to 60 degrees Celsius and pH 4.0 to pH 13.0. In vitro antimicrobial tests revealed that the optical density (OD) value of the bacterial culture added to the phage was consistently lower than that of the phage-free culture, highlighting a significant inhibitory effect. No known resistance, lysogenicity, or virulence-related genes were detected. It can be used as a reference isolate for *E. coli* phages and a source of biological materials and data support for research on bacteriophage-related treatment of porcine *E. coli*.

## Figures and Tables

**Table 1:** Phage Psq-1 gene prediction and functional annotation.

Functional Module	ORF	Function	Identity/%	E Value	Accession Number
Lysis	3	holin-like class II protein	98.28%	2.00 × 10^−139^	NC_073088.1
	4	holin-like class I protein	97.55%	1.00 × 10^−112^	NC_016566.1
	5	lysozyme	95.53%	0	NC_016566.1
Structural and packaging	9	terminase	100.00%	7.00 × 10^−138^	YP_004957437.1
	10	terminase large subunit	93.58%	0	NC_024783.1
	11	minor tail protein	99.80%	0	YP_010741994.1
	16	major head protein	99.73%	0	YP_008550148.1
	19	tail protein	94.94%	0	NC_024783.1
	21	major tail protein	97.52%	0	NC_073088.1
	25	minor tail protein	98.49%	8.00 × 10^−143^	YP_010741450.1
	26	minor tail protein	99.24%	0	YP_009291470.1
	27	tail assembly protein	98.37%	0	YP_009288147.1
	28	tail assembly protein	99.02%	1.00 × 10^−144^	YP_010742501.1
	29	tail fiber tip protein	98.52%	0	YP_010742348.1
DNA replication and regulatory	24	tail-length tape-measure protein	99.09%	0	YP_004957454.1
	37	DNA methylase	97.52%	9.00 × 10^−115^	YP_010741570.1
	38	DNA ligase	81.58%	8.00 × 10^−81^	YP_009055311.1
	40	DNA cytosine methyltransferase	99.57%	5.00 × 10^−171^	YP_010741369.1
	41	helicase	99.37%	0	YP_010742490.1
	43	holiday junction resolvase	100.00%	5.00 × 10^−59^	XDN94337.1
	44	DNA polymerase	99.08%	0	YP_002720046.1
	48	exonuclease	97.69%	0	YP_010741426.1
	52	DNA recombination nuclease inhibitor gamma	100.00%	4.00 × 10^−72^	YP_010741422.1
	53	helicase-primase	99.20%	0	WRQ05368.1
Metabolism	7	DNA repair exonuclease	98.68%	0.00 × 10^0^	WYA83814.1
	39	HNH endonuclease protein	98.13%	4.00 × 10^−72^	YP_010741962.1
Unknown functional protein	12	head morphogenesis protein	96.74%	0	NC_016566.1
Hypothetical protein	1	hypothetical protein	91.19%	0	NC_073067.1
	2	hypothetical protein	97.25%	4.00 × 10^−153^	ON548431.1
	6	hypothetical protein	98.26%	0	NC_019724.1
	8	hypothetical protein	98.12%	2.00 × 10^−98^	LN881730.1
	13	hypothetical protein	97.87%	1.00 × 10^−157^	NC_019419.2
	14	hypothetical protein	93.20%	0	NC_027383.1
	15	hypothetical protein	100%	0	YP_010741704.1
	17	hypothetical protein	99.27%	0	NC_073079.1
	18	hypothetical protein	97.18%	2.00 × 10^−166^	NC_024783.1
	20	hypothetical protein	89.34%	8.00 × 10^−146^	NC_016566.1
	22	hypothetical protein	100%	2.00 × 10^−83^	YP_010741944.1
	23	hypothetical protein	97.89%	1.00 × 10^−36^	NC_019724.1
	30	hypothetical protein	100%	0	YP_010742349.1
	31	hypothetical protein	97.26%	2.00 × 10^−44^	YP_010740694.1
	32	hypothetical protein	83.85%	8.00 × 10^−146^	XEN42225.1
	33	hypothetical protein	98.43%	6.00 × 10^−137^	YP_010742352.1
	34	hypothetical protein	97.10%	6.00 × 10^−123^	YP_010741505.1
	35	hypothetical protein	100%	3.00 × 10^−113^	YP_010741823.1
	36	hypothetical protein	94.00%	2.00 × 10^−24^	YP_010740538.1
	39	hypothetical protein	99.07%	1.00 × 10^−72^	YP_007112638.1
	42	hypothetical protein	97.30%	5.00 × 10^−47^	YP_010741367.1
	45	hypothetical protein	98.21%	1.00 × 10^−30^	YP_010740637.1
	46	hypothetical protein	89.43%	2.00 × 10^−166^	YP_010741971.1
	47	hypothetical protein	98.70%	1.00 × 10^−50^	WRQ05362.1
	49	hypothetical protein	98.39%	2.00 × 10^−38^	YP_002720051.1
	50	hypothetical protein	98.80%	3.00 × 10^−52^	YP_002720052.1
	51	hypothetical protein	98.24%	1.00 × 10^−105^	YP_010742534.1
	54	hypothetical protein	96.55%	3.00 × 10^−32^	YP_010741020.1
	55	hypothetical protein	96.04%	2.00 × 10^−64^	XEC67447.1

## Data Availability

All data and materials are contained in this article.
